# Shared Decision-Making and Patient Decision Aids for Percutaneous Left Atrial Appendage Occlusion

**DOI:** 10.1001/jamanetworkopen.2025.56937

**Published:** 2026-02-13

**Authors:** Joshua B. Rager, Chien-Yu Huang, Sarah Zimmerman, Sanket S. Dhruva, James V. Freeman, Daniel D. Matlock, Karl Minges, Amneet Sandhu, Erica S. Spatz, Paul Varosy, Tanner J. Caverly

**Affiliations:** 1 Division of General Internal Medicine and Geriatrics, Department of Medicine, Indiana University School of Medicine, Indianapolis; 2 Center for Health Services Research, Regenstrief Institute, Indianapolis, Indiana; 3 Center for Outcomes Research and Evaluation, Yale New Haven Health Services Corporation, New Haven, Connecticut; 4 Section of Cardiology, Department of Medicine, University of California, San Francisco, School of Medicine, San Francisco; 5 Philip R. Lee Institute for Health Policy Studies, University of California, San Francisco, School of Medicine, San Francisco; 6 Section of Cardiovascular Medicine, Yale University School of Medicine, New Haven, Connecticut; 7 Division of Geriatric Medicine, Department of Medicine, University of Colorado School of Medicine, Aurora; 8 Veterans Affairs (VA) Eastern Colorado Geriatric Research Education and Clinical Center, Denver; 9 Division of Cardiovascular Medicine, Section of Electrophysiology, University of Colorado School of Medicine, Aurora; 10 Eastern Colorado VA Healthcare System, Aurora; 11 Department of Learning Health Sciences, University of Michigan Medical School, Ann Arbor; 12 Center for Clinical Management Research, Ann Arbor Veterans Affairs Medical Center, Ann Arbor, Michigan

## Abstract

**Importance:**

Experts recommend a shared decision-making (SDM) process before percutaneous left atrial appendage occlusion (pLAAO) in patients with atrial fibrillation, and the Centers for Medicare & Medicaid Services (CMS) require SDM with the use of a patient decision aid (DA) as a condition for reimbursement. However, little is known about how these guidelines and policies have influenced practice.

**Objective:**

To describe overall trends in reported SDM and use of DAs for pLAAO and to identify key patient, operator, and institutional factors associated with their use.

**Design, Setting, and Participants:**

This cohort study analyzed data from October 1, 2022, to June 30, 2024, from the American College of Cardiology’s National Cardiovascular Data Registry (NCDR) LAAO Registry. Participants included patients who underwent first-time pLAAO.

**Exposure:**

pLAAO implantation.

**Main Outcomes and Measures:**

The proportion of encounters reporting SDM and DA use (SDM plus DA) overall and each month. Hierarchical logistic regression was used to estimate the odds of reported SDM plus DA, the probability of SDM plus DA, and the variance in SDM plus DA associated with operator and institutional levels.

**Results:**

A total of 147 296 unique patient encounters (86 593 [58.8%] male; mean [SD] age, 76.6 [7.7] years) were included. Of 830 institutions participating in the NCDR LAAO Registry during the study period, 829 (99.9%) reported on SDM and 817 (98.4%) reported on DA use. In the unadjusted analysis, 95 305 encounters (64.7%) reported SDM plus DA had occurred. Unadjusted rates of SDM plus DA rose steadily during the study period from 62.5% in October 2022 to 75.0% in June 2024. The adjusted analysis suggests that the observed variance in SDM plus DA reporting was large and attributable primarily to the institutional level (median odds ratio, 115.64; 95% CI, 79.71-151.56). The range of estimated probability of SDM plus DA by institution was 0.1% to 76.4%, with a mean (SD) of 52.0% (28.6%). There was no statistically significant difference in odds of SDM plus DA for patients with Medicare vs those without (odds ratio, 1.03; 95% CI, 0.98-1.09).

**Conclusions and Relevance:**

In this cohort study of patients who underwent pLAAO, SDM plus DA reporting was high, but there was large variation between institutions. Patients with Medicare did not have greater odds of reported SDM plus DA, despite the CMS requirement. These findings exhibit the need for further exploration of institutional barriers and facilitators to SDM and DA use for pLAAO.

## Key Points

**Question:**

What are the key features associated with reported shared decision-making (SDM) and use of patient decision aids (DAs) for percutaneous left atrial appendage occlusion (pLAAO) in patients with atrial fibrillation?

**Findings:**

In this cohort study of 147 296 patients who received pLAAO, the observed variance in SDM plus DA reporting was large and was attributable primarily to the institutional level.

**Meaning:**

These findings suggest that whether a patient is reported as having been engaged in SDM and received a DA before pLAAO depends more on the institution performing the procedure than any other factor, underscoring the need for systems-level research to better support patient-centered care.

## Teaser

This cohort study describes the use of shared decision-making and decision aids and identifies key patient, operator, and institutional factors associated with their use among patients undergoing percutaneous left atrial appendage occlusion for atrial fibrillation.

## Introduction

In 2023, the American College of Cardiology–American Heart Association Joint Committee on Clinical Practice Guidelines strengthened their recommendation for the use of percutaneous left atrial appendage occlusion (pLAAO) in patients with atrial fibrillation (AF) at elevated risk of stroke who have nonreversible contraindications to long-term anticoagulation therapy. Additionally, they added a new recommendation for pLAAO in patients with AF at high bleeding risk after a discussion about risks, benefits, and consideration of patient preferences.^1^

Important uncertainties remain about the comparative effectiveness of pLAAO to oral anticoagulation and the long-term outcomes of pLAAO.^2^ Given the need for careful patient selection and a focus on the risk-benefit tradeoffs in this decision, engaging patients in an explicit shared decision-making (SDM) process before pLAAO is an expert consensus recommendation.^3^ Furthermore, the Centers for Medicare & Medicaid Services (CMS) have required SDM and the use of a patient decision aid (DA) as a condition of reimbursement since 2016.^4,5^

SDM and the use of DAs have been shown to increase patient knowledge and improve decision-making for various cardiovascular diseases^6^ and many other disease states.^7^ Despite current guidelines and CMS policies emphasizing the importance of SDM and DAs for pLAAO, there is very little published evidence describing their use in clinical practice^8^ and none to describe national trends. To address this gap, we sought to understand the use of SDM and DAs for pLAAO on a national level by describing trends in SDM and DA use as reported in the American College of Cardiology’s National Cardiovascular Data Registry (NCDR) and to identify key patient, operator, and institutional factors associated with SDM and DA use.

## Methods

### Data Source

The NCDR LAAO registry has been described previously.^9^ In brief, the LAAO Registry is approved by CMS to meet the registry requirement as outlined in the national coverage determination for pLAAO. The NCDR has an established data quality reporting process that includes annual random audits to ensure data completeness, accuracy and validity.^10^ During the most recent audit of the LAAO registry reported in January 2025, there was a 94% agreement rate between registry-reported data with source document review and 100% agreement between registry-reported and billing data.

Since October 2022, the NCDR has included separate data elements for SDM and DA use as a part of its standard data collection for the LAAO Registry. Data for the NCDR LAAO Registry are collected and submitted by the reporting hospital sites, primarily through medical record review by abstractors. Specifically, for the SDM element, site coders are instructed to provide a yes or no answer to the prompt “indicate if shared decision making was performed for the procedure” and separately, for the DA element, provide a yes or no answer to “indicate if a shared decision making tool [DA] was used.”

The Yale University Human Investigation Committee approved analysis of a limited dataset derived from the LAAO Registry with a waiver of informed consent owing to the use of deidentified data. The study adhered to the Strengthening the Reporting of Observational Studies in Epidemiology (STROBE) reporting guideline for cohort studies.

### Cohort Definition

After excluding data that did not pass an initial NCDR quality check, we identified all patients 18 years or older who underwent pLAAO from October 1, 2022, to June 30, 2024. We excluded those encounters where the SDM or DA element were missing (n = 983 [<1% of encounters in the study period]). In the event of multiple pLAAO procedures for an individual patient, we included only the first instance where pLAAO was performed.

### Outcomes

Our primary analysis examined SDM plus DA, defined as encounters in which both SDM and DA use were reported as occurring in the standard data collection form. Our secondary analysis examined SDM reporting that could further include reported DA use but also captures encounters where SDM alone occurred (ie, no DA use reported).

### Statistical Analysis

We first examined unadjusted use of SDM plus DA and SDM alone by month and by insurance payer source to describe general trends in the NCDR. If multiple insurance types were identified for an individual and one of these was Medicare or Medicare Advantage, we categorized those patients as Medicare patients. We also examined the unadjusted distribution of SDM plus DA and SDM alone across institutions.

We then used 3-level hierarchical logistic regression to estimate the odds of SDM plus DA among patient, operator, and institutional covariates. Covariates were determined based on the clinical and research expertise of the coauthors and prior literature that has identified relevant factors influencing DA and SDM use in other areas.^11,12,13,14,15^ This was further supplemented by stepwise logistic regression (eMethods in Supplement 1).

The covariates included in the final model were age, sex, body mass index, race and ethnicity, Medicare (vs not), CHA_2_DS_2_VASC (congestive heart failure, hypertension, aged ≥75 years, diabetes, stroke or transient ischemic attack [TIA], vascular disease, aged 65-74 years, and female sex) score, HAS-BLED (uncontrolled hypertension, abnormal kidney or liver function, history of stroke, history of major bleeding, labile international normalized ratio, aged >65 years, and medication use predisposing to bleeding or excessive alcohol use) score, AF classification, stroke history, TIA, prior thromboembolic event, sleep apnea, and indication for occlusion. Operator variables included procedural volume; institutional level variables included institution type, teaching status, region, setting, and procedural volume. Details on race and ethnicity data collection and reporting are provided in the eMethods in Supplement 1); categories included Asian, Black, White, and other race (including American Indian or Alaska Native and Native Hawaiian or Other Pacific Islander) and Hispanic or non-Hispanic ethnicity. Patients were able to select multiple categories. These data were collected to understand whether SDM or DA reporting varied significantly across race and ethnicity categories.

We then estimated the variance in SDM plus DA explained by the operator and institutional levels through calculating a median odds ratio (MOR) and intraclass correlation (ICC), first in a model without covariates (empty model) and then with the covariates added. The MOR represents the median value of all possible ORs when comparing 2 randomly selected individuals from different clusters. Put differently, the MOR is the median value of the OR between the institutions and operators at the lowest and the highest probability of SDM plus DA.^16,17^ An MOR greater than 1.00 indicates that moving an individual from one low-performing cluster to another high-performing cluster would increase the odds of that individual achieving the outcome. The ICC provides an additional quantitative measure for assessing the amount of clustering present in the data. The same process was also performed to examine reported SDM as well. We then used the empty 3-level model to estimate the probabilities of DA-based SDM and SDM per institution. Further model descriptions are presented in the eMethods in Supplement 1). Statistical significance for all ORs was set at a 2-sided *P* < .05. Statistical analyses were performed with the use of SAS, version 9.4 (SAS Institute Inc).

## Results

A total of 830 institutions participated in the registry during the study period. Of those, 829 institutions (99.9%) reported on SDM and 817 (98.4%) reported on DA use for 147 296 unique patient encounters. The mean (SD) age for patients in the cohort was 76.6 (7.7) years; 60 703 (41.2%) were female and 86 593 (58.8%) were male. The mean (SD) body mass index (calculated as weight in kilograms divided by height in meters squared) was 29.8 (8.6). In terms of race and ethnicity, 1721 patients (11.6%) were Asian, 5336 (3.6%) were Black; 136 865 (92.9%) were White, and 3448 (2.3%) were other race; 5310 (3.6%) were Hispanic. A total of 96 010 patients (65.2%) had Medicare coverage. The mean (SD) CHA_2_DS_2_VASC score was 4.66 (1.48) and the mean (SD) HAS-BLED score was 2.72 (1.11). A complete list of the patient, operator, and institutional characteristics for the cohort is provided in eTable 1 in Supplement 1.

Of the 147 296 included encounters, 132 797 (90.2%) reported SDM alone and 95 305 (64.7%) reported SDM plus DA. Patient, operator, and institutional characteristics for SDM plus DA are presented in Table 1. The patient, operator, and institutional characteristics for SDM are presented in eTable 2 in Supplement 1.

**Table 1.** Patient, Operator, and Facility Characteristics by Reporting of SDM and DA Use

Characteristic	SDM plus DA (n = 95 305)	SDM without DA (n = 37 492)	No SDM or DA (n = 14 499)
**Patients**				
Age, mean (SD), y	76.6 (7.7)	76.6 (7.8)	76.5 (8.0)
Sex, No. (%)			
Male	55 747 (58.5)	22 177 (59.2)	8669 (59.8)
Female	39 558 (41.5)	15 315 (40.8)	5830 (40.2)
BMI, mean (SD)	29.7 (9.0)	29.8 (8.6)	29.5 (8.6)
CHA_2_DS_2_-VASc score, mean (SD)	4.66 (1.5)	4.67 (1.5)	4.63 (1.5)
HAS-BLED score, mean (SD)	2.79 (1.1)	2.60 (1.1)	2.56 (1.1)
Hemoglobin level, mean (SD), g/dL	12.92 (2.0)	12.85 (2.0)	12.78 (2.0)
Albumin level, mean (SD), g/dL	3.93 (0.5)	3.95 (0.5)	3.97 (0.5)
Race, No. (%)			
Asian	1009 (1.1)	450 (1.2)	262 (1.8)
Black	3370 (3.5)	1307 (3.5)	659 (4.5)
White	88 812 (93.2)	34 911 (93.1)	13 142 (90.6)
Other race^a^	2164 (2.3)	837 (2.2)	447 (3.1)
Hispanic ethnicity, No. (%)	3264 (3.4)	1339 (3.6)	707 (4.9)
Comorbid conditions and medical history, No. (%)			
Congestive heart failure	33 613 (35.3)	13 335 (35.6)	5138 (35.4)
Hypertension	86 949 (91.2)	34 005 (90.7)	13 038 (89.9)
Diabetes	32 032 (33.6)	12 725 (33.9)	4755 (32.8)
Stroke	17 685 (18.6)	7408 (19.8)	2944 (20.3)
Prior thromboembolic event	10 262 (10.8)	3807 (10.2)	1697 (11.7)
Vascular disease	48 667 (51.1)	18 972 (50.6)	7036 (48.5)
Abnormal kidney function	11 980 (12.6)	4862 (13.0)	1711 (11.8)
Abnormal liver function	2458 (2.6)	1134 (3.0)	408 (2.8)
Alcohol use	4883 (5.1)	1918 (5.1)	699 (4.8)
Attempt at AF termination	44 460 (46.7)	17 100 (45.6)	6638 (45.8)
Cardiomyopathy	17 710 (18.6)	6590 (17.6)	2691 (18.6)
Chronic lung disease	17 905 (18.8)	7109 (19.0)	2683 (18.5)
Coronary artery disease	40 208 (42.2)	15 640 (41.7)	5816 (40.1)
Sleep apnea	30 702 (32.2)	11 602 (30.9)	4359 (30.1)
CKD stage 3 or greater (eGFR <60)	37 120 (38.9)	14 454 (38.6)	5617 (38.7)
AF classification, No. (%)^b^			
Paroxysmal	61 335 (64.4)	23 803 (63.5)	8983 (62.0)
Persistent	17 974 (18.9)	7554 (20.1)	3097 (21.4)
Long-standing persistent	5704 (6.0)	2045 (5.5)	892 (6.2)
Permanent	9584 (10.1)	3774 (10.1)	1381 (9.5)
Valvular	164 (0.2)	99 (0.3)	46 (0.3)
Insurance payer, No. (%)^c^			
Private	47 533 (49.9)	17 465 (46.6)	7469 (51.5)
Medicare	61 684 (64.7)	24 378 (65.0)	9948 (68.6)
Medicaid	4115 (4.3)	1900 (5.1)	792 (5.5)
State-specific plan	625 (0.7)	155 (0.4)	111 (0.8)
Other	22 121 (23.2)	8743 (23.3)	2781 (19.2)
Indication for occlusion, No. (%)			
Increased thromboembolic risk	61 323 (64.3)	24 129 (64.4)	9097 (62.7)
History of major bleed	37 552 (39.4)	14 908 (39.8)	5634 (38.9)
High fall risk	40 731 (42.7)	15 478 (41.3)	5476 (37.8)
Labile INR	2446 (2.6)	760 (2.0)	277 (1.9)
Patient preference	42 152 (44.2)	16 083 (42.9)	5568 (38.4)
Nonadherence with anticoagulation therapy	4297 (4.5)	1593 (4.2)	676 (4.7)
Clinically significant bleeding risk	27 964 (29.3)	9721 (25.9)	3332 (23.0)
**Operator and hospital**				
Annual operator procedure volume, No. (%)			
Low (first quartile: 1-12)	3518 (3.7)	1547 (4.1)	1181 (8.1)
Medium low (second quartile: 13-22)	10 884 (11.4)	4788 (12.8)	2141 (14.8)
Medium high (third quartile: 23-38)	22 574 (23.7)	9647 (25.7)	3688 (25.4)
High (fourth quartile: 39-111)	58 329 (61.2)	21 510 (57.4)	7489 (51.7)
Type of facility, No. (%)			
Private or community	82 783 (86.9)	31 013 (82.7)	11 200 (77.2)
University	10 467 (11.0)	6077 (16.2)	3113 (21.5)
Government	2055 (2.2)	402 (1.1)	186 (1.3)
Teaching hospital	48 283 (50.7)	20 855 (55.6)	9778 (67.4)
Region, No. (%)^d^			
Northeast	9533 (10.0)	6125 (16.3)	3434 (23.7)
West	18 600 (19.5)	6552 (17.5)	3308 (22.8)
Midwest	22 891 (24.0)	10 403 (27.7)	3541 (24.4)
South	44 207 (46.4)	14 405 (38.4)	4153 (28.6)
Location of facility, No. (%)			
Urban	56 586 (59.4)	21 536 (57.4)	9203 (63.5)
Suburban	29 216 (30.7)	12 772 (34.1)	4074 (28.1)
Rural	9503 (10.0)	3184 (8.5)	1222 (8.4)
Annual procedural volume at institution, No. (%)			
Low (first quartile: 1-40)	5290 (5.6)	2521 (6.7)	872 (6.0)
Medium low (second quartile: 41-65)	13 003 (13.6)	7138 (19.0)	2774 (19.1)
Medium high (third quartile: 66-103)	26 332 (27.6)	9734 (26.0)	3195 (22.0)
High (fourth quartile: 104-300)	50 680 (53.2)	18 099 (48.3)	7658 (52.8)

Abbreviations: AF, atrial fibrillation; BMI, body mass index (calculated as weight in kilograms divided by height in meters squared); CHA_2_DS_2_-VASc, congestive heart failure, hypertension, aged ≥75 years, diabetes, stroke or transient ischemic attack [TIA], vascular disease, aged 65-74 years, and female sex; CKD, chronic kidney disease; DA, decision aid; eGFR, estimated glomerular filtration rate; HAS-BLED, uncontrolled hypertension, abnormal kidney or liver function, history of stroke, history of major bleeding, labile international normalized ratio, aged >65 years, and medication use predisposing to bleeding or excessive alcohol use; INR, international normalized ratio; SDM, shared decision-making.

SI conversion factors: To convert albumin to g/L, multiply by 10; hemoglobin to g/L, multiply by 10.

^a^ Includes American Indian or Alaska Native and Native Hawaiian or Other Pacific Islander. Patients could select multiple races.

^b^ Not specified if missing data.

^c^ Categories are not mutually exclusive.

^d^ Institution region could be missing if not enough information was found in data to locate region.

**Figure 1.**
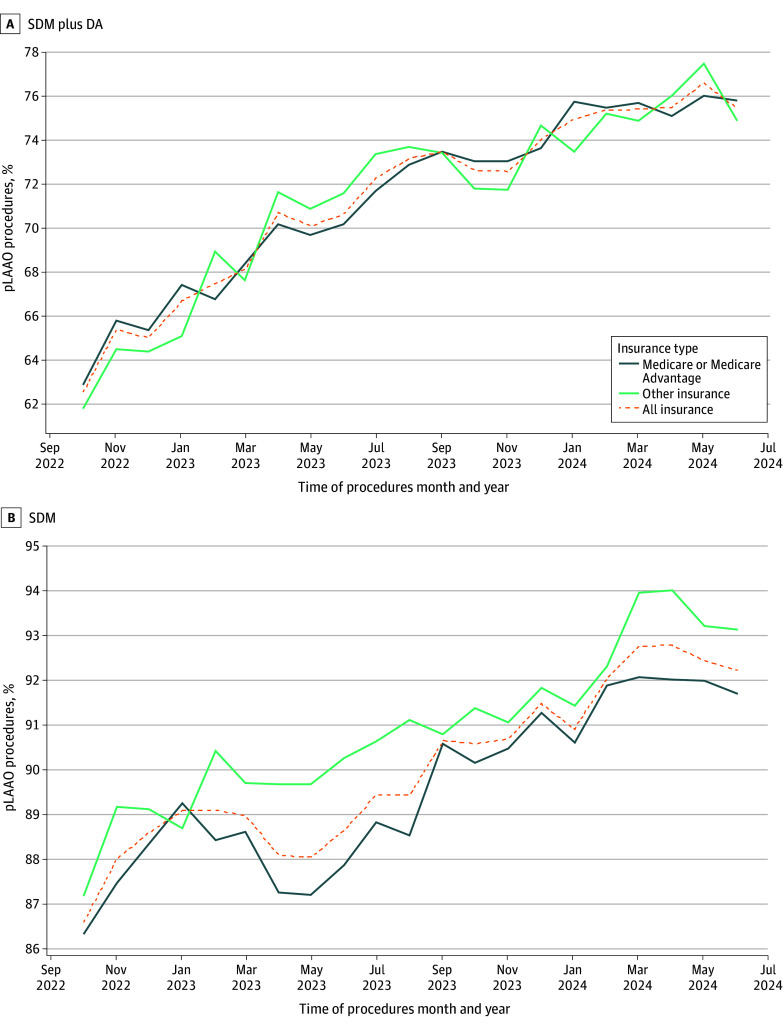
Proportion of Percutaneous Left Atrial Appendage Occlusion (pLAAO) Procedures With Reported Shared Decision-Making (SDM) Plus Decision Aid (DA) Use and SDM Alone Data are stratified by patient insurer.

Figure 1 shows the temporal trends in SDM plus DA and SDM alone during the study period by insurer category. The overall unadjusted proportion of reported SDM plus DA increased from 62.5% in October 2022 to 75.5% in June 2024. The overall unadjusted proportion of reported SDM increased from 86.5% to 92.2% during this time. For patients with Medicare, SDM plus DA rates increased from 63.0% in the first month to 75.8% in the last month of the study period.

**Figure 2.**
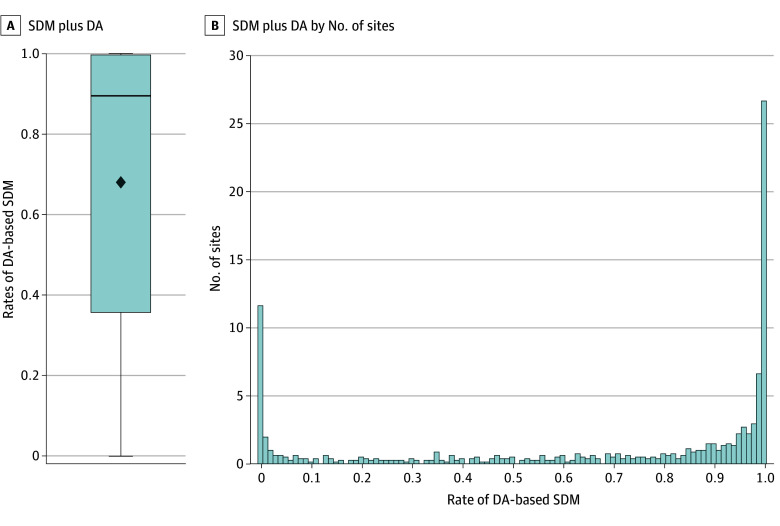
Distribution of Reported Shared Decision-Making (SDM) Plus Decision Aid (DA) Use by Institution A, Box and whisker plot of SDM plus DA (mean [SD], 68.0% [38.3%]; median, 89.5% [IQR, 35.7%-99.6%]). B, Histogram of the rate of SDM plus DA and the number of sites reporting at that rate; some institutions (1.4%) never report SDM plus DA, whereas some (3.3%) always report SDM plus DA.

Figure 2 and eFigure 1 in Supplement 1 show the distribution of SDM plus DA and SDM alone use across all sites, respectively. The mean (SD) for SDM plus DA use was 68.0% (38.3%) with a median of 89.5% (IQR, 35.7%-99.6%). The mean (SD) for SDM was 89.1% (21.6%) with a median of 98.8% (IQR, 91.3%-100%).

### Adjusted Analyses

#### SDM Plus DA

Table 2 shows the estimated odds ratios for SDM plus DA. The ICC for the empty model was 0.89, indicating that 89% of the observed variation in SDM plus DA can be explained by differences between clusters of operators and institutions. After covariates were added, the ICC was 0.88; the final model estimated the operator level variance at 1.13 (SE, 0.08) and institutional level variance at 23.66 (SE, 1.65), meaning that the larger contribution of the observed variation in SDM plus DA can be attributed to differences at the institution level specifically. The MOR was 115.64 (95% CI, 79.71-151.56), meaning the odds of reported SDM plus DA for a patient in the highest vs the lowest performing clusters was 115 times greater, at median.

**Table 2.** Adjusted Analyses of SDM Plus DA Use Reporting and SDM Only Reporting

Measure of association	SDM plus DA, OR (95% CI)	*P* value	SDM alone, OR (95% CI)	*P* value
**Individual-level variables**				
Age, y				
<65	1 [Reference]	NA	1 [Reference]	NA
66-70	1.02 (0.92-1.14)	.56	1.03 (0.92-1.16)	.79
71-75	1.00 (0.91-1.11)	.50	1.04 (0.93-1.16)	.86
76-80	1.05 (0.95-1.17)	.25	1.07 (0.96-1.19)	.59
81-85	1.12 (1.01-1.25)	.04	1.12 (1.01-1.25)	.04
>85	1.14 (1.01-1.28)	.02	1.03 (0.91-1.16)	.10
Sex				
Female	1 [Reference]	NA	1 [Reference]	NA
Male	0.99 (0.94-1.04)	.31	0.97 (0.92-1.02)	.36
BMI				
<18.5	0.86 (0.71-1.04)	.11	0.8 (0.66-0.97)^a^	.02
18.5-24.9	0.98 (0.92-1.04)	.37	0.94 (0.88-1.00)	.37
25.0-29.9	1 [Reference]	NA	1 [Reference]	NA
30.0-34.9	0.98 (0.93-1.05)	.18	0.96 (0.89-1.02)	.70
35.0-39.9	0.95 (0.88-1.03)	.74	0.98 (0.90-1.07)	.27
≥40.0	0.96 (0.87-1.06)	.63	1.02 (0.91-1.14)	.50
Race				
Black	1.12 (0.99-1.27)	.55	1.05 (0.93-1.20)	.64
White	1 [Reference]	NA	1 [Reference]	NA
Other^a^	1.23 (1.08-1.39)	.02	0.98 (0.86-1.11)	.59
Hispanic ethnicity	1 (0.87-1.14)	.88	0.91 (0.80-1.04)	.16
Medicare or MA insurance	1.03 (0.98-1.09)	.12	0.94 (0.89-0.99)	.048
CHA_2_DS_2_VASC score				
≤2	1 [Reference]	NA	1 [Reference]	NA
2 to <4	0.98 (0.88-1.11)	.14	1.09 (0.97-1.22)	.14
4 to <6	0.95 (0.83-1.07)	.48	1.05 (0.92-1.19)	.16
≥6	0.89 (0.76-1.03)	.52	0.95 (0.81-1.11)	.99
HAS-BLED score				
≤2	1 [Reference]	NA	1 [Reference]	NA
2 to <3	1.14 (1.08-1.21)	<.001	1.14 (1.07-1.21)	<.001
3 to <4	1.21 (1.13-1.30)	<.001	1.21 (1.11-1.31)	<.001
4 to <5	1.25 (1.11-1.41)	<.001	1.29 (1.12-1.48)	<.001
≥5	1.52 (1.16-2.00)	<.001	1.17 (0.86-1.59)	.30
Normal hemoglobin level	1.05 (1.01-1.10)	.03	1.06 (1.01-1.11)	.03
AF classification				
Paroxysmal	1 [Reference]	NA	1 [Reference]	NA
Persistent	0.94 (0.88-0.99)	.02	1.06 (0.99-1.13)	.17
Long-standing persistent	1.14 (1.02-1.27)	.002	1.01 (0.90-1.12)	.99
Permanent	0.98 (0.90-1.05)	.97	1.00 (0.92-1.09)	.92
Medical history				
Stroke	0.88 (0.81-0.95)	.003	0.97 (0.90-1.05)	.44
TIA	0.96 (0.89-1.04)	.72	0.98 (0.90-1.07)	.58
Prior thromboembolic event	1.04 (0.95-1.13)	.11	0.87 (0.80-0.95)	.02
Sleep apnea	1.06 (1.01-1.12)	.01	1.03 (0.97-1.09)	.35
Indication for occlusion				
Increased thromboembolic risk	1.26 (1.19-1.35)	<.001	1.28 (1.19-1.36)	<.001
High fall risk	1.06 (1.01-1.12)	<.001	1.26 (1.19-1.33)	<.001
Labile INR	1.06 (0.91-1.24)	.28	1.12 (0.93-1.34)	.28
Patient preference	0.97 (0.91-1.03)	.31	1.08 (1.01-1.16)	.004
Clinically meaningful bleeding risk	1.11 (1.05-1.17)	<.001	1.28 (1.20-1.36)	<.001
**Operator-level variables**				
Operator volume				
Low (first quartile)	1 [Reference]	NA	1 [Reference]	NA
Medium low (second quartile)	0.99 (0.77-1.27)	.55	1.23 (0.95-1.59)	.09
Medium high (third quartile)	0.99 (0.77-1.26)	.63	1.2 (0.94-1.54)	.10
High (fourth quartile)	0.96 (0.75-1.24)	.30	1.51 (1.16-1.95)	<.001
**Institutional-level variables**				
Type of facility				
Private	0.87 (0.04-20.43)	.38	1.26 (0.17-9.18)	.37
Government	0.94 (0.34-2.60)	.76	1.8 (0.93-3.48)	.77
University	1 [Reference]	NA	1 [Reference]	NA
Teaching hospital	0.53 (0.24-1.17)	.41	1.05 (0.65-1.69)	.32
Procedural volume				
Low	1 [Reference]	NA	1 [Reference]	NA
Medium low (second quartile)	0.56 (0.19-1.65)	.26	0.69 (0.35-1.38)	.27
Medium high (third quartile)	1.34 (0.45-3.95)	.72	1.38 (0.70-2.73)	.66
High (fourth quartile)	0.8 (0.27-2.37)	.13	1.14 (0.58-2.24)	.11
Region				
South	1 [Reference]	NA	1 [Reference]	NA
Midwest	0.29 (0.12-0.75)	.01	0.89 (0.50-1.59)	.28
Northeast	0.09 (0.03-0.27)	<.001	0.37 (0.18-0.75)	<.001
West	0.61 (0.22-1.67)	.28	0.59 (0.32-1.10)	.69
Suburban hospital (vs urban)	1.59 (0.78-3.23)	.19	1.02 (0.64-1.65)	.12
Rural hospital	0.46 (0.18-1.19)	.11	1.23 (0.59-2.55)	.11
**Measures of variation or clustering**				
Operator level variance, mean (SE)	1.13 (0.08)	NA	1.39 (0.09)	NA
Institutional level variance, mean (SE)	23.66 (1.65)	NA	9.35 (0.72)	NA
Median OR (95% CI)	115.64 (79.71-151.56)	<.001	22.78 (18.00-27.56)	<.001
ICC	0.88	NA	0.77	NA

Abbreviations: AF, atrial fibrillation; BMI, body mass index; CHA_2_DS_2_-VASc, congestive heart failure, hypertension, aged ≥75 years, diabetes, stroke or transient ischemic attack [TIA], vascular disease, aged 65-74 years, and female sex; DA, decision aid; HAS-BLED, uncontrolled hypertension, abnormal kidney or liver function, history of stroke, history of major bleeding, labile international normalized ratio, aged >65 years, and medication use predisposing to bleeding or excessive alcohol use; ICC, intraclass correlation; INR, international normalized ratio; OR, odds ratio; SDM, shared decision-making.

^a^ Includes American Indian or Alaska Native and Native Hawaiian or Other Pacific Islander.

Compared with patients 65 years or younger, patients aged 66 to 70 years (OR, 1.02; 95% CI, 0.92-1.14), 71 to 75 years (OR, 1.00; 95% CI, 0.91-1.11), and 76 to 80 years (OR, 1.05; 95% CI, 0.95-1.17) had similar odds of SDM plus DA use, while patients aged 81 to 85 years (OR, 1.12; 95% CI, 1.01-1.25) and older than 85 years (OR, 1.14; 95% CI, 1.01-1.28) had higher odds. There were no significant differences between sexes, White and Black patients, or Hispanic and non-Hispanic patients, although patients of other race or ethnicity had higher odds of SDM plus DA compared with their White counterparts (OR, 1.23; 95% CI, 1.08-1.39). There was no statistically significant difference in SDM plus DA between those patients insured by Medicare and those not (OR, 1.03; 95% CI, 0.98-1.09).

While no statistical differences were observed across CHA_2_DS_2_VASC scores, higher HAS-BLED scores were associated with higher odds of SDM plus DA (Table 2). Compared with patients who had paroxysmal AF, those with persistent AF had lower odds of SDM plus DA use (OR, 0.94; 95% CI, 0.88-0.99), while those with long-standing persistent AF had higher odds of SDM plus DA (OR, 1.14; 95% CI, 1.02-1.27). Those with a documented history of stroke were less likely to have SDM plus DA compared with those without prior stroke (OR, 0.88; 95% CI, 0.81-0.95).

**Figure 3.**
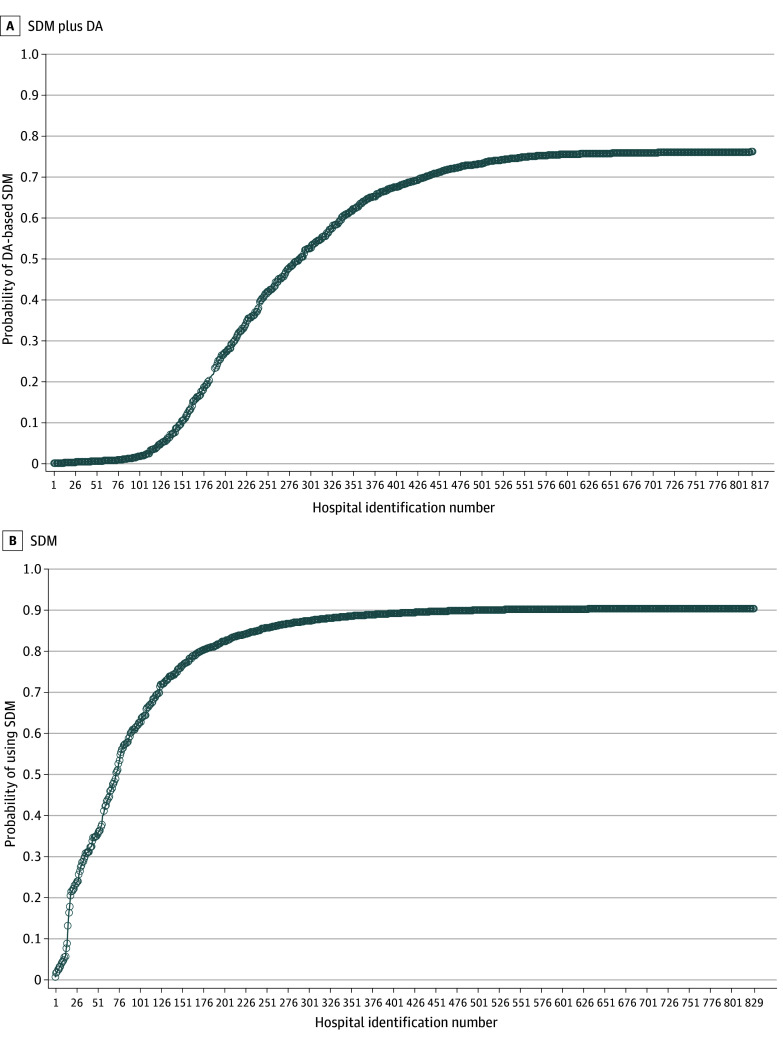
Probability of Reported Shared Decision-Making (SMD) Plus Decision Aid (DA) Use and SDM Alone by Institution Hospital identification numbers are ordered by probability of DA-based SDM and SDM alone. Each circle represents the estimated probability for each institution and is calculated from the empty model. A, Probability of DA-based SDM (mean [SD], 52.0% [28.6%]; median, 68.1% [IQR, 28.0%-75.6%]). B, Probability of SDM alone (mean [SD], 81.2% [18.7]; median, 89.6% [IQR, 83.1%-90.6%]).

Regarding documented indication for pLAAO and compared with patients without these indications, patients with reported increased thromboembolic risk (OR, 1.26; 95% CI, 1.19-1.35), high fall risk (OR, 1.06; 95% CI, 1.01-1.12), and clinically significant bleeding risk (OR, 1.11; 95% CI, 1.05-1.17) had greater odds of SDM plus DA. There were no statistically significant differences observed for patient preference (OR, 0.97; 95% CI, 0.91-1.03). The mean (SD) estimated probability of DA-based SDM was 52.0% (28.6%); median, 68.1% (IQR, 28.0%-75.6%); and range, 0.1% to 76.4% (Figure 3).

#### SDM Alone

Table 2 shows the estimated odds for SDM alone. In the empty model, 77% of the observed variation in SDM can be explained by differences between clusters of operators and institutions. After covariates were added, the final model estimated the operator level variance at 1.39 (SE, 0.09), institutional level variance at 9.35 (SE, 0.72), MOR at 22.78 (95% CI, 18.00-27.56), and ICC at 0.77.

Compared with patients 65 years or younger, patients aged 81 to 85 years had higher odds of SDM alone (OR, 1.12; 95% CI, 1.01-1.25), with other age-groups having similar odds to those 65 years or younger (Table 2). No statistically significant differences were observed among sex and race and ethnicity. Patients with Medicare had lower odds of SDM alone (OR, 0.94; 95% CI, 0.89-0.99).

Those with higher HAS-BLED scores generally had higher odds of SDM alone (Table 2). No statistically significant differences were observed according to AF classification or prior stroke, or TIA. Prior thromboembolic event was associated with lower odds of SDM alone (OR, 0.87; 95% CI, 0.80-0.95).

Regarding documented indication for pLAAO and compared with patients without these indications, patients with reported increased thromboembolic risk (OR, 1.28; 95% CI, 1.19-1.36), high fall risk (OR, 1.26; 95% CI, 1.19-1.33), and clinically significant bleeding risk (OR, 1.28; 95% CI, 1.20-1.36) had greater odds of SDM alone. Those for whom the indication included patient preference had higher odds of SDM (OR, 1.08; 95% CI, 1.01-1.16). Operators in the highest quartile of procedural volume had greater odds of SDM alone (OR, 1.51; 95% CI, 1.16-1.95). The mean (SD) estimated probability of SDM alone by site was 81.2% (18.7%), the median was 89.6% (IQR, 83.2%-90.6%), and the range was 0.8% to 90.8% (Figure 3).

## Discussion

In this national cohort study, we found that reported SDM plus DA and SDM alone for pLAAO was high in the NCDR and increased during the study period, suggesting that guidelines and policy emphasizing SDM and DA use have had a strong influence on clinical practice. However, there is considerable variation in these outcomes across institutions. Surprisingly, whether a patient is reported to have undergone SDM and been presented with a DA is much better explained by knowing at which institution the patient is having the procedure than any patient or operator characteristic we identified. To our knowledge, this is the first study in any disease state to produce quantitative estimates of clinical variation in SDM and DA use attributable to the institutional level. Understanding this variation is critical for identifying gaps in SDM and DA implementation, informing policy and quality improvement efforts, and ultimately ensuring more consistent and patient-centered decision-making across institutions.

Since pLAAO was first covered by CMS in 2016, reimbursement has been contingent on both SDM and DA use.^5^ We found that compared with all other insurers, being insured by Medicare did not increase the odds of SDM plus DA, and in fact, among patients for whom SDM alone was reported, having Medicare resulted in statistically significant lower odds of reported SDM, although this difference was small. These findings are likely in part due to the high rates of SDM alone and SDM plus DA we observed for all patients, regardless of insurer. We hypothesize that one explanation for this is that the typical referral, operational, and clinical care pathways for advanced procedures such as pLAAO needed to be restructured to accommodate the DA-based SDM requirement and so it would be impractical for hospitals and physician groups to have different pathways for patients covered by different insurers. Significant operational changes and resource investments have been seen in other areas where DA-based SDM has been required by the CMS,^18,19^ such as implantable cardioverter-defibrillators for the primary prevention of sudden cardiac death in patients with low ejection fraction^20^ and lung cancer screening.^21^ The capacity and resources needed to adhere to this policy may have disproportionately impacted institutions that treat more patients with Medicare coverage.

In a recently published survey of physicians who perform pLAAO,^8^ 41% indicated that policies about SDM processes in their practice were set by the physician group or practice, 36% by the hospital or hospital system, and 23% by the implanting physician. Further, approximately one-half of respondents indicated that SDM occurred during a separate visit before the preprocedural consultation, whereas the other half reported that SDM occurred during the preprocedural consultation.^8^ These findings suggest substantial differences in the local implementation of this policy and may further explain why we observed significant variability among institutions.

While reported SDM plus DA for all patients and those with Medicare was high in our study, it was not 100%. Despite years of research in the development and evaluation of DAs in a wide range of disease states, their implementation into the clinical environment has not yet been realized and is an area of continued need and ongoing research.^22^ At least 1 DA for pLAAO is available online,^23^ but despite the CMS mandate to perform DA-based SDM with an “evidence-based tool,” we are not aware of any study that has assessed the efficacy of a DA for pLAAO to improve decision-making. Future SDM and DA policy should consider this reality of clinical practice and consider providing further resources to evaluate the barriers and facilitators to DA implementation at varying institutions, disseminate best practices, and consider additional incentives to promote DA use and uptake.

SDM is most relevant for preference-sensitive medical decisions where there is medical uncertainty about the best option, and so patient values and informed preferences are needed to know which course of action is best.^24,25,26,27^ We found that about 40% of encounters indicated patient preference as a reason for implantation, and this increased the odds of reported SDM use, although not reported SDM plus DA. However, we also found that SDM plus DA and SDM alone were reported more often when the indication to undergo pLAAO appeared more clinically clear than not. For example, we found that the odds of reported SDM plus DA and SDM alone generally increased as HAS-BLED scores increased and that those with reported indications such as high fall risk and clinically meaningful bleeding risk were associated with greater odds of reported SDM plus DA and SDM alone. Paradoxically, the rationale for directly recommending pLAAO in these individuals would appear to be more substantiated by recent evidence and guidelines and could reasonably make it less likely for a SDM encounter to be needed.^1^

This paradox may be explained by how clinicians sometimes use DAs. Rather than primarily supporting patient autonomy in preference-sensitive situations, clinicians may be more likely to use DAs to reinforce what they believe is the best decision, such as recommending pLAAO when a patient has a clear indication. While SDM is often framed as most necessary when there is clinical uncertainty, our findings suggest that in practice, SDM and DA-based SDM may function more as tools for risk communication and patient education rather than as mechanisms for weighing trade-offs in equipoise.^28^ This aligns with prior research showing that clinicians do not always distinguish between SDM and informed decision-making.^29,30^

We did not observe any racial, ethnic, or sex differences in adjusted analyses. Rates of SDM use in other areas have shown key disparities along these lines and training, and use of SDM and DAs has shown some potential to lessen these disparities.^6^ We hypothesize that one potential advantage of mandated SDM and DA use is that it may make it more likely to be uniformly applied to all patients within an institution.

### Limitations

This study has some limitations, including coding for SDM and DA use, which is largely based on data from medical record abstraction. The sensitivity and specificity for these data elements in capturing SDM and DA use as idealized in a clinical encounter is unknown. However, there is no consensus on a gold standard for measuring SDM.^31^ Furthermore, our results showed variation in reporting within hospitals as well, which suggests there is intrainstitution discrimination between cases where SDM and DA use is and is not occurring. The most recent data quality audit in the LAAO registry reported 100% agreement between registry-reported data and Medicare claims; because the CMS will only reimburse pLAAO if there is documentation of SDM and DA use, this further supports the validity of these variables. The NCDR LAAO Registry is not mandatory for all institutions that perform pLAAO, and thus a selection bias may exist; however, the registry is the only CMS-approved registry and is the first and largest database for pLAAO reporting. Furthermore, all patients in our cohort had pLAAO, and therefore we are unable to capture those who may have undergone DA-based SDM but ultimately did not have pLAAO. As with all observational research, there is the potential for additional, unmeasured confounding; for instance, the registry does not capture who performed the SDM encounter, and this could represent another level of potential variance.

## Conclusions

In this cohort study of patients receiving pLAAO in the NCDR, we found that reported DA-based SDM and SDM alone used for pLAAO varies significantly between institutions. Further system-level research and interventions are necessary to address the barriers and facilitators of DA-based SDM.

## Notes

Supplement 1. **eMethods.** Model Description, Stepwise Regression, and Race and Ethnicity Reporting **eTable 1.** Patient, Operator, and Institutional Characteristics of Included Cohort (N = 147 296) **eTable 2.** Patient, Operator, and Facility Characteristics by Reporting of Shared Decision-Making Alone **eFigure.** Distribution of Reported Shared Decision-Making Alone by Institution

Supplement 2.
Data Sharing Statement
