# Supplementary material for: Shared Decision-Making and Patient Decision Aids for Percutaneous Left Atrial Appendage Occlusion
Source: JAMA Netw Open. 2026 Feb 13;9(2):e2556937. doi: 10.1001/jamanetworkopen.2025.56937 (PMC12905655; doi:10.1001/jamanetworkopen.2025.56937)
Supplement: Supplement 1. — eMethods. Model Description, Stepwise Regression, and Race and Ethnicity Reporting eTable 1. Patient, Operator, and Institutional Characteristics of Included Cohort (N = 147 296) eTable 2. Patient, Operator, and Facility Characteristics by Reporting of Shared Decision-Making Alone eFigure. Distribution of Reported Shared Decision-Making Alone by Institution [file jamanetwopen-e2556937-s001.pdf]

Supplementary Online Content

Rager JB, Huang CY, Zimmerman S, et al. Shared decision-making and patient decision aids for percutaneous left atrial appendage occlusion. *JAMA Netw Open.* 2026;9(2):e2556937. doi:10.1001/jamanetworkopen.2025.56937

eMethods. Model Description, Stepwise Regression, and Race and Ethnicity Reporting

eTable 1. Patient, Operator, and Institutional Characteristics of Included Cohort (N = 147 296)

eTable 2. Patient, Operator, and Facility Characteristics by Reporting of Shared Decision-Making Alone

eFigure. Distribution of Reported Shared Decision-Making Alone by Institution

This supplementary material has been provided by the authors to give readers additional information about their work.

© 2026 Rager JB et al. *JAMA Network Open*.

eMethods. Model Description, Stepwise Regression, and Race and Ethnicity Reporting

Model description:

The hierarchical logistic regression model includes random intercepts at both the operator and institutional levels to account for clustering of patients within operators and operators within institutions. The model was estimated using the Laplace approximation in SAS PROC GLIMMIX. The corresponding statistical model can be expressed as:

$$\text{logit}(P(Y_{ijk} = 1)) = \beta_o + \beta^T X_{ijk} + u_k + v_{jk}$$

where Y_{ijk} denotes the binary outcome for patient i treated by operator j in institution k, X_{ijk} represents the vector of patient-, operator-, and institutional-level covariates, $u_k \sim N(0, \sigma^2\text{institution})$ is the random intercept for institution k, and $v_{jk} \sim N(0, \sigma^2\text{operator})$ is the random intercept for operator j nested within institution k. β^T represents the transpose of the coefficient matrix (i.e., $\beta^T X_{ijk} = [\beta_1 X_{1ijk} + \beta_2 X_{2ijk} + \cdots + \beta_p X_{pijk}]$

Thus, the model includes random intercepts only (no random slopes), reflecting variability in the baseline log-odds of the outcome across operators and institutions. Each patient was assigned to one operator, and each operator was assigned to one institution where they conducted the majority of their procedures.

Model diagnostics: For model diagnostics, we reviewed the convergence criteria, the Goodness of fit test, AUC, and checked correlations for multicollinearity. We are assuming the random effects are independent of the fixed effects. Additionally, we reviewed the Q-Q plots of the random intercepts to confirm that the random effects were normally distributed and plotted the residuals vs predicted values to assess the residuals.

Handling of missing data: We did not include continuous variables in the analysis that are missing more than 5%. For a continuous variable missing less than 5%, then we assign the missing value to be equal to the median. If the missing variable is binary, then we assume the missing value to be 0, “no”.

Stepwise regression:

Variables were included in the stepwise regression if they had a p-value <0.1 in univariate analysis. The significance level to be included in the final model was 0.05. Variables entered in stepwise logistic regression for selection included: age, body-mass index (BMI), sex, race/ethnicity, Medicare (vs not), CHA_2DS_2VASC score, HASBLED score, hypertension, diabetes, stroke, transient ischemic attack (TIA), prior thromboembolic event, vascular disease, abnormal renal function, valvular atrial fibrillation, coronary artery disease, sleep apnea, atrial fibrillation classification (paroxysmal, persistent, long standing persistent, permanent), and reported indication for pLAAO (increased thromboembolic risk, history of major bleed, high fall risk, labile INR, patient preference, clinically significant bleeding risk). Operator level variables included annual procedural volume within the NCDR divided into quartiles. Institution level variables included institution type (private vs university vs government), teaching hospital (vs non-teaching), US region, setting (urban vs suburban vs rural), and annual procedural volume at the institution divided into quartiles. Once variables were selected, they were included in the multi-level analysis. Stepwise logistic regression was performed once for all variable selection. The same variables were used for both hierarchical models.

Race and ethnicity reporting:

LAAO registry site abstractors and coders are instructed to report patient race and ethnicity as determined by the patient or family. Race categories in the LAAO registry include White (defined as individuals with origins in any of the original peoples of Europe, including, for example, English, German, Irish, Italian, Polish, and Scottish); Black/African American(defined as Individuals with origins in any of the Black racial groups of Africa, including, for example, African

© 2026 Rager JB et al. *JAMA Network Open*.

American, Jamaican, Haitian, Nigerian, Ethiopian, and Somali); American Indian/ Alaska Native (defined as Individuals with origins in any of the original peoples of North, Central, and South America, including, for example, Navajo Nation, Blackfeet Tribe of the Blackfeet Indian Reservation of Montana, Native Village of Barrow Inupiat Traditional Government, Nome Eskimo Community, Aztec, and Maya); Asian (defined as Individuals with origins in any of the original peoples of Central or East Asia, Southeast Asia, or South Asia, including, for example, Chinese, Asian Indian, Filipino, Vietnamese, Korean, and Japanese); Native Hawaiian/Pacific Islander (defined as individuals with origins in any of the original peoples of Hawaii, Guam, Samoa, or other Pacific Islands, including, for example, Native Hawaiian, Samoan, Chamorro, Tongan, Fijian, and Marshallese).

For this analysis those identifying as American Indian/ Alaska Native or Native Hawaiian/Pacific Islander were classified as "Other" race. Ethnicity included: Hispanic/Latino(defined as individuals of Mexican, Puerto Rican, Salvadoran, Cuban, Dominican, Guatemalan, and other Central or South American or Spanish culture or origin) or Not. These classifications are based on the U.S. Office of Management and Budget, Classification of Federal Data on Race and Ethnicity during October 2022 when the NCDR LAAO registry data collection form and data dictionary were last updated.

eResults.

eTable 1. Patient, Operator, and Institutional Characteristics of Included Cohort (N = 147 296)

**Patient characteristics**	
Age, years [Mean (SD)]	76.62 (7.73)
Male (n, %)	86593 (58.79%)
BMI (kg/m2), [Mean (SD)]	29.77 (8.62)
CHA2DS2-VASc Score [Mean (SD)]	4.66 (1.48)
HAS-BLED Score [Mean (SD)]	2.72 (1.11)
Hemoglobin (mean, SD)	12.89 (1.98)
Albumin (Mean, SD)	3.94 (0.47)
**Race/Ethnicity (n, %)**	
Asian	1721 (1.17%)
Black	5336 (3.62%)
White	136865 (92.92%)
Other race*	3448 (2.34%)
Hispanic ethnicity	5310 (3.60%)
**Co-morbid conditions and medical history (n, %)**	
Congestive heart failure	52086 (35.36%)
Hypertension	133992 (90.97%)
Diabetes	49512 (33.61%)
Stroke	28037 (19.03%)
Prior thromboembolic event	15766 (10.70%)
Vascular disease	74675 (50.70%)
Abnormal renal function	18553 (12.60%)
Abnormal liver function	4000 (2.72%)
Alcohol use	7500 (5.09%)
Attempt at AF termination	68198 (46.30%)
Cardiomyopathy	26991 (18.32%)
Chronic lung disease	27697 (18.80%)
Coronary artery disease	61664 (41.86%)
Sleep apnea	46663 (31.68%)
CKD3 or greater (eGFR less than 60)	57191 (38.83%)
**Atrial fibrillation classification (n, %)**	
Paroxysmal	94121 (63.90%)
Persistent	28625 (19.43%)

© 2026 Rager JB et al. *JAMA Network Open*.

Long standing persistent	8641 (5.87%)
Permanent	14739 (10.01%)
Valvular atrial fibrillation	309 (0.21%)
**Insurance payer(s) (n,%)**	
Private	72467 (49.20%)
Medicare	96010 (65.18%)
Medicaid	6807 (4.62%)
State-Specific plan	891 (0.60%)
Other	33645 (22.84%)
**Indication for occlusion (n, %)**	
Increased thromboembolic risk	94549 (64.19%)
History of major bleed	58094 (39.44%)
High fall risk	61685 (41.88%)
Labile INR	3483 (2.36%)
Patient preference	63803 (43.32%)
Non-compliance with anticoagulation therapy	6566 (4.46%)
Clinically significant bleeding risk	41017 (27.85%)
**Annual operator procedure volume (n, %)**	
low (1st quartile): 1-12	6246 (4.24%)
medium low (2nd quartile): 13-22	17813 (12.09%)
medium high (3rd quartile): 23-38	35909 (24.38%)
high (4th quartile): 39-111	87328 (59.29%)
**Type of facility (n, %)**	
Private or community	124996 (84.86%)
University	19657 (13.35%)
Government	2643 (1.79%)
Teaching hospital	78916 (53.58%)
**Region (n, %)**	
Northeast	19092 (12.96%)
West	28460 (19.32%)
Midwest	36835 (25.01%)
South	62765 (42.61%)
**Location of facility (n, %)**	
Urban	87325 (59.29%)
Suburban	46062 (31.27%)
Rural	13909 (9.44%)
**Annual procedural volume at institution (n,%)**	
low (1st quartile): 1-40	8683 (5.89%)
medium low (2nd quartile): 41-65	22915 (15.56%)
medium high (3rd quartile): 66-103	39261 (26.65%)
high (4th quartile): 104-300	76437 (51.89%)

* Other race includes those identifying as American Indian/ Alaska Native or Native Hawaiian/Pacific Islander

© 2026 Rager JB et al. *JAMA Network Open*.

**eTable 2.** Patient, Operator, and Facility Characteristics by Reporting of Shared Decision-Making Alone		
	SDM Alone (n=132,797)	no SDM (14,499)
**Patient characteristics**		
Age, years [Mean (SD)]	76.63 (7.7)	76.47 (8.0)
Male (n, %)	77,924 (58.7%)	8,669 (59.8%)
Female (n, %)	54,939 (41.3%)	5,830 (40.2%)
BMI (kg/m2), [Mean (SD)]	29.80 (8.6)	29.50 (8.6)
CHA2DS2-VASc Score [Mean (SD)]	4.67 (1.5)	4.63 (1.5)
HAS-BLED Score [Mean (SD)]	2.74 (1.1)	2.56 (1.1)
Hemoglobin (mean, SD)	12.90 (2.0)	12.78 (2.0)
Albumin (Mean, SD)	3.93 (0.5)	3.97 (0.5)
**Race/Ethnicity (n, %)**		
Asian	1,459 (1.1%)	262 (1.8%)
Black	4,677 (3.5%)	659 (4.6%)
White	123,723 (93.2%)	13,142 (90.6%)
Other race*	3,001 (2.3%)	447 (3.1%)
Hispanic ethnicity	4,603 (3.5%)	707 (4.9%)
**Co-morbid conditions and medical history (n, %)**		
Congestive heart failure	46,948 (35.4%)	5,138 (35.4%)
Hypertension	120,954 (91.1%)	13,038 (89.9%)
Diabetes	44,757 (33.7%)	4,755 (32.8%)
Stroke	25,093 (18.9%)	2,944 (20.3%)
Prior thromboembolic event	14,069 (10.6%)	1,697 (11.7%)
Vascular disease	67,639 (50.9%)	7,036 (48.5%)
Abnormal renal function	16,842 (12.7%)	1,711 (11.8%)
Abnormal liver function	3,592 (2.7%)	408 (2.8%)
Alcohol use	6,801 (5.1%)	699 (4.8%)
Attempt at AF termination	61,560 (46.4%)	6,638 (45.8%)
Cardiomyopathy	24,300 (18.3%)	2,691 (18.6%)
Chronic lung disease	25,014 (18.8%)	2,683 (18.5%)
Coronary artery disease	55,848 (42.1%)	5,816 (40.1%)
Sleep apnea	42,304 (31.9%)	4,359 (30.1%)
CKD3 or greater (eGFR less than 60)	51,574 (38.8%)	5,617 (38.7%)
**Atrial fibrillation classification (n, %)**		
Paroxysmal	85,138 (64.1%)	8,983 (62.0%)
Persistent	25,528 (19.2%)	3,097 (21.4%)
Long standing persistent	7,749 (5.8%)	892 (6.2%)
Permanent	13,358 (10.1%)	1,381 (9.5%)
Valvular atrial fibrillation	263 (0.2%)	46 (0.3%)
**Insurance payer(s) (n,%)**		
Private	64,998 (49.0%)	7,469 (51.5%)
Medicare	86,062 (64.8%)	9,948 (68.6%)
Medicaid	6,015 (4.5%)	792 (5.5%)
State-Specific plan	780 (0.6%)	111 (0.8%)
Other	30,864 (23.2%)	2,781 (19.2%)
**Indication for occlusion (n, %)**		
Increased thromboembolic risk	85,452 (64.4%)	9,097 (62.7%)
History of major bleed	52,460 (39.5%)	5,634 (38.9%)
High fall risk	56,209 (42.3%)	5,476 (37.8%)

© 2026 Rager JB et al. *JAMA Network Open*.

Labile INR	3,206 (2.4%)	277 (1.9%)
Patient preference	58,235 (43.9%)	5,568 (38.4%)
Non-compliance with anticoagulation therapy	5,890 (4.4%)	676 (4.7%)
Clinically significant bleeding risk	37,685 (28.4%)	3,332 (23.0%)
**Annual operator procedure volume (n, %)**		
low (1st quartile): 1-12	5,065 (3.8%)	1,181 (8.2%)
medium low (2nd quartile): 13-22	15,672 (11.8%)	2,141 (14.8%)
medium high (3rd quartile): 23-38	32,221 (24.3%)	3,688 (25.4%)
high (4th quartile): 39-111	79,839 (60.1%)	7,489 (51.7%)
**Type of facility (n, %)**		
Private or community	113,796 (85.7%)	11,200 (77.3%)
University	16,544 (12.5%)	3,113 (21.5%)
Government	2,457 (1.9%)	186 (1.3%)
Teaching hospital	69,138 (52.1%)	9,778 (67.4%)
**Region (n, %)**		
Northeast	15,658 (11.8%)	3,434 (23.7%)
West	25,152 (18.9%)	3,308 (22.8%)
Midwest	33,294 (25.1%)	3,541 (24.4%)
South	58,612 (44.1%)	4,153 (28.6%)
**Location of facility (n, %)**		
Urban	78,122 (58.8%)	9,203 (63.5%)
Suburban	41,988 (31.6%)	4,074 (28.1%)
Rural	12,687 (9.6%)	1,222 (8.4%)
**Annual procedural volume at institution (n,%)**		
low (1st quartile): 1-40	7,811 (5.9%)	872 (6.0%)
medium low (2nd quartile): 41-65	20,141 (15.2%)	2,774 (19.1%)
medium high (3rd quartile): 66-103	36,066 (27.2%)	3,195 (22.0%)
high (4th quartile): 104-300	68,779 (51.8%)	7,658 (52.8%)

* Other race includes those identifying as American Indian/ Alaska Native or Native Hawaiian/Pacific Islander

© 2026 Rager JB et al. *JAMA Network Open*.

eFigure. Distribution of Reported Shared Decision-Making Alone by Institution

Panel A.

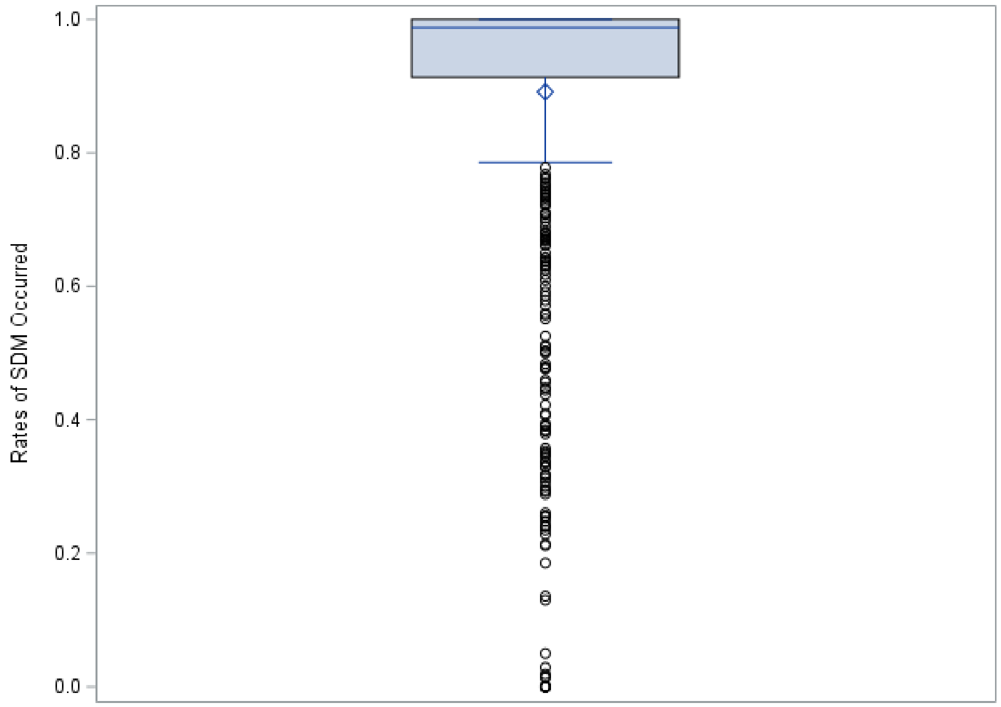

Panel B.

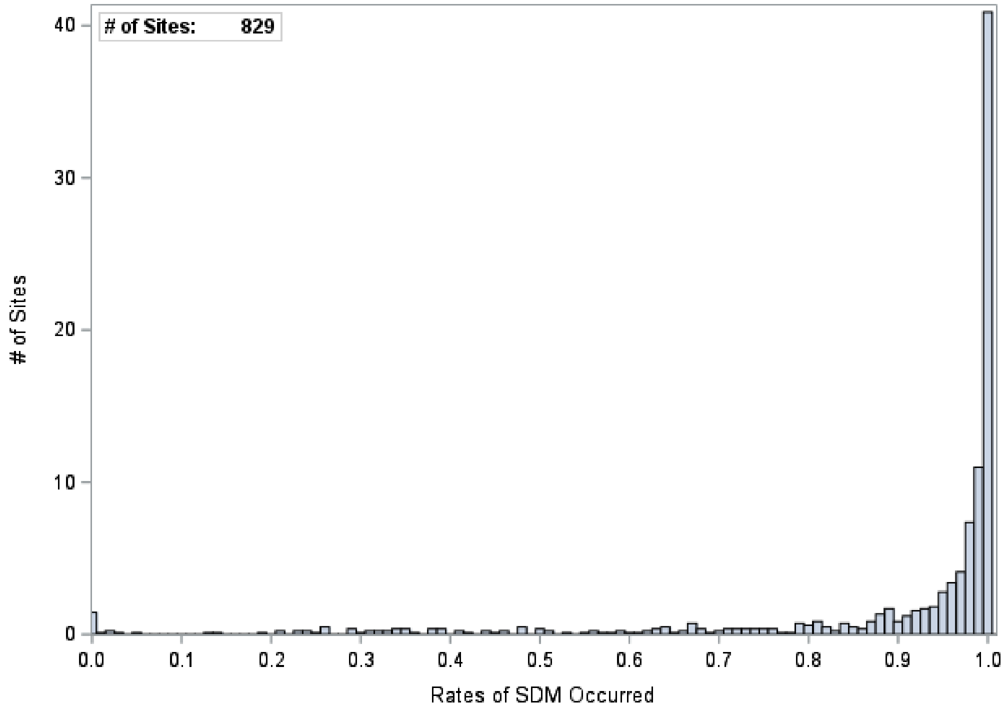

Legend. Distribution of SDM alone by institution. Panel A presents a box-and-whisker plot and Panel B presents a histogram of the rate of SDM alone and the number of sites reporting at that rate. Compared to SDM+DA, fewer sites report never performing SDM alone.

© 2026 Rager JB et al. *JAMA Network Open*.
