# Supplementary material for: Shared Decision-Making and Patient Decision Aids for Percutaneous Left Atrial Appendage Occlusion
Source: JAMA Netw Open. 2026 Feb 13;9(2):e2556937. doi: 10.1001/jamanetworkopen.2025.56937 (PMC12905655; doi:10.1001/jamanetworkopen.2025.56937)
Supplement: Supplement 2. — Data Sharing Statement [file jamanetwopen-e2556937-s002.pdf]

Data Sharing Statement

Rager. Shared Decision-Making and Patient Decision Aids for Percutaneous Left Atrial Appendage Occlusion. *JAMA Netw Open*. Published February 13, 2026.
doi:10.1001/jamanetworkopen.2025.56937

Data
Data available: No

Additional Information
Explanation for why data not available: Data is only available through the NCDR
